# Skeletal Muscle Metastases to the Flexor Digitorum Superficialis and Profundus from Urothelial Cell Carcinoma and Review of the Literature

**DOI:** 10.1155/2016/2387501

**Published:** 2016-08-28

**Authors:** Marco Guidi, Cesare Fusetti, Stefano Lucchina

**Affiliations:** ^1^Hand Surgery Unit, General Surgery Department, Hospital La Carità, Locarno, Switzerland; ^2^Hand Surgery Unit, General Surgery Department, Hospital San Giovanni Bellinzona, Bellinzona, Switzerland

## Abstract

Urothelial cell carcinoma (UCC) metastases to skeletal muscle are extremely rare and usually found in patients with advanced stage cancer. The most common sites of bladder cancer metastases are lymph nodes, lung, liver, and bones. Muscle is an unusual site of metastases from a distant primary cancer, due to several protective factors. We present a rare case of 76-year-old patient with metastases in the flexor digitorum superficialis (FDS) and flexor digitorum profundus (FDP) muscles, 2 years after a radical cystectomy for invasive UCC of the bladder. This case is the first description of a forearm lesion, with an extensive infiltration of the volar compartments of the forearm, and the first one with a clear functional impairment.

## 1. Introduction

UCC is the most common primary malignancy of the urinary tract, accounting for more than 90% of all bladder cancers, and may affect its entire length, from renal pelvis to bladder [1]. UCC is usually a tumor of older patients, with the average age of presentation of 65 years, with a strong male predilection (M : F = 4 : 1) [1].

Skeletal muscle metastases from bladder cancer are very rare. In the English literature, only five studies reported [2–6] on skeletal muscle metastases from UCC with clinical-radiological findings.

The case that we present is the first report of metastases involving the FDS and FDP muscles after an invasive UCC of the bladder.

The authors have obtained the patient's informed written consent for print and electronic publication of the case report.

## 2. Case Report

A 76-year-old male presented to the general practitioner with gross hematuria and urinary frequency. The ultrasound (US) imaging and the cystoscopy revealed a mass in the bladder. A transurethral resection was undertaken and an invasive carcinoma (T3, N0, and M0) was found at the histological examination. Three cycles of neoadjuvant chemotherapy were performed with cisplatin and gemcitabine.

Four months later, a radical cystectomy and an ileal conduit urinary diversion were performed. 1 year after, a complete thoracoabdominal computed tomography (CT) was negative for metastases.

18 months later, the patient presented a persistent anterior right forearm pain, resistant to analgesic, at first underestimated by the general practitioner and treated as tendonitis.

**Figure 1:**
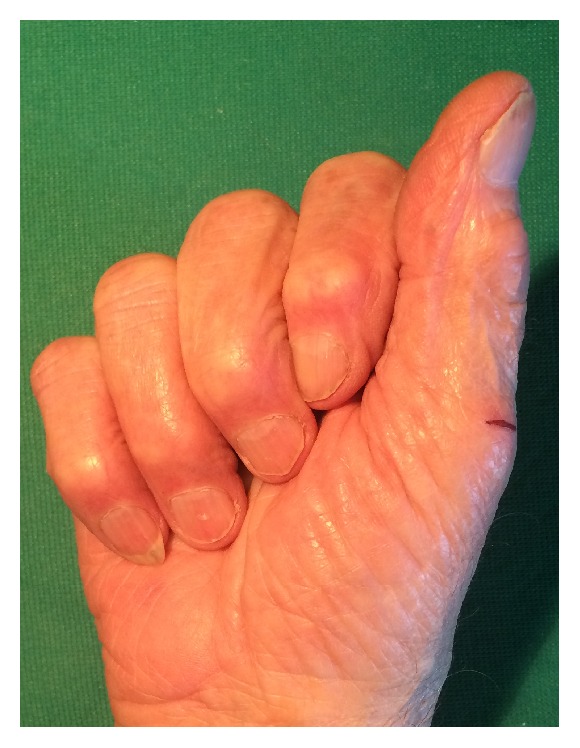
Clinical presentation of the flexion contracture of the fingers of the right hand.

The patient was referred to our hand surgery unit six months later for persistent pain of the right forearm and a painful flexion contracture of the proximal interphalangeal joints of the fingers (Figure 1) with the clinical suspicion of chronic compartment syndrome.

The consequent clenched fist position was not passively reducible. Spasticity was also supposed, but there was no evidence of hyperactive response to quick stretch of the long fingers [7].

**Figure 2:**
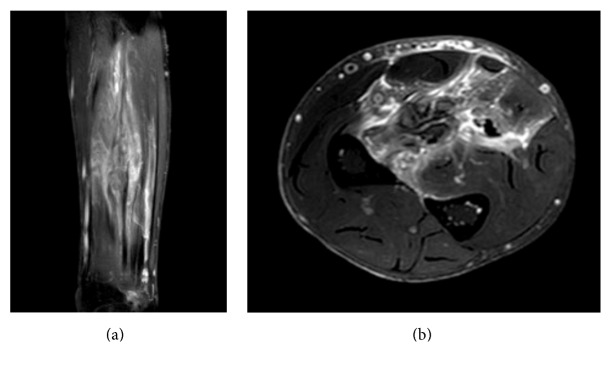
MRI of the right forearm ((a), coronal view; (b), axial view): infiltrating lesion of the superficial and deep volar compartment of the forearm with a widespread edema.

The Magnetic Resonance Imaging (MRI) revealed an irregular ring-enhancing lesion of 7.5 cm × 2.7 cm × 2.1 cm in the superficial and deep volar compartment of the forearm (Figure 2). The lesion was infiltrating the muscular body of the FDS and FDP.

**Figure 3:**
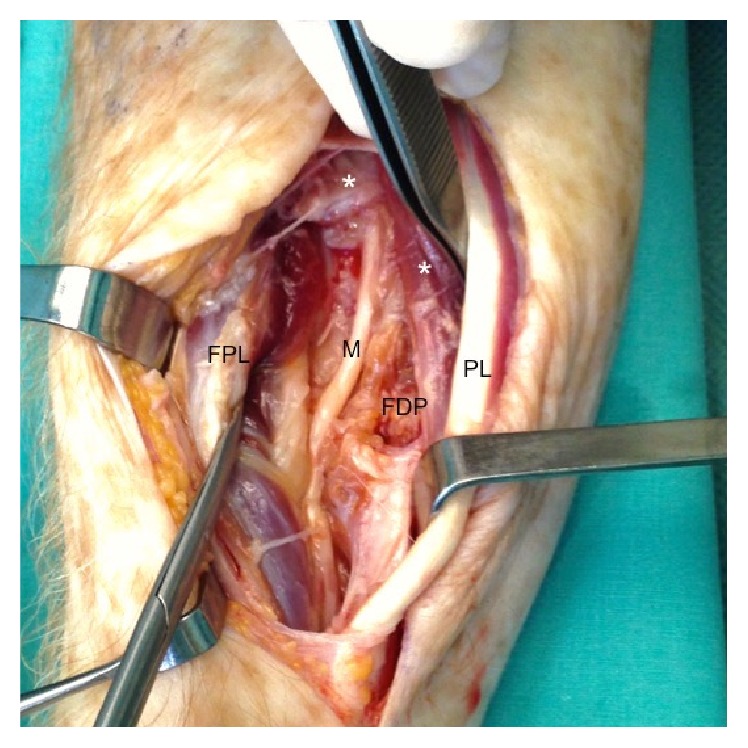
Intraoperative findings. *∗*: Flexor digitorum superficialis partially resected; PL: palmaris longus; FPL: flexor pollicis longus; M: median nerve; FDP: flexor digitorum profundus.

A surgical explorative excision (Figure 3) of the lesion was performed with a resection en bloc of the FDS of the long fingers. The deep infiltration of the FDP and partially of the median nerve avoided a radical primary resection.

**Figure 4:**
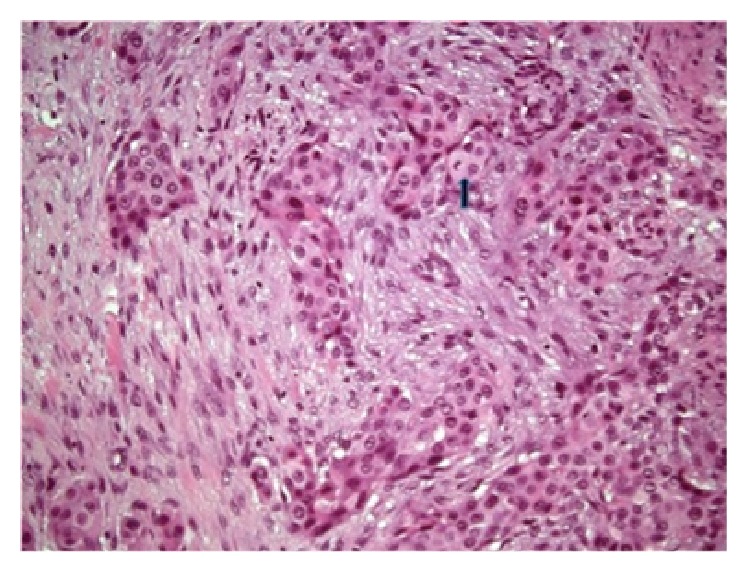
Nests of polygonal cells surrounded by stroma reaction of transitional cell carcinoma (arrow: mitotic figure), higher magnification. H&E ×200.

**Figure 5:**
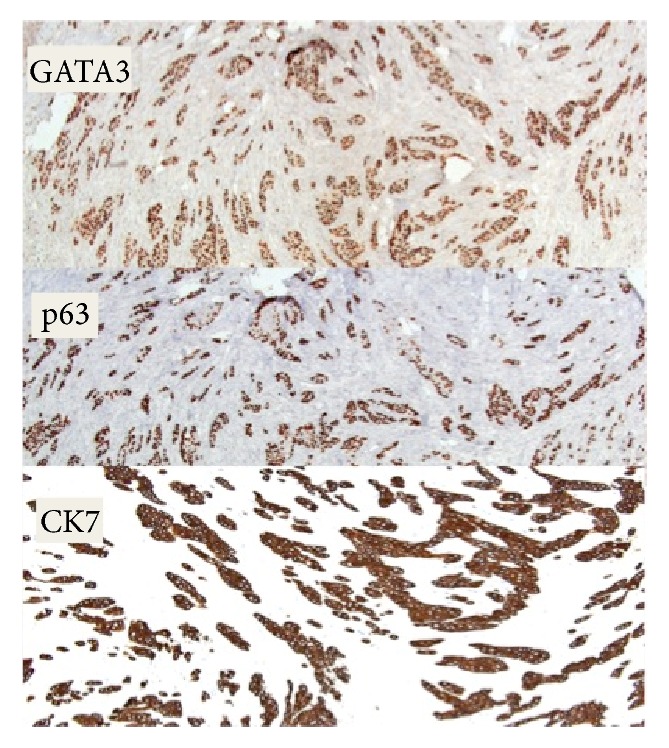
Immunohistochemistry: CK7, p63, and GATA3 expression in malignant cells, consistent with transitional cell differentiation.

The pathology reported a skeletal muscle metastasis compatible with a high grade UCC positive to the CK7, p63, GATA3, and AE1/AE3 markers and negative to the CK20 (Figures 4 and 5).

**Figure 6:**
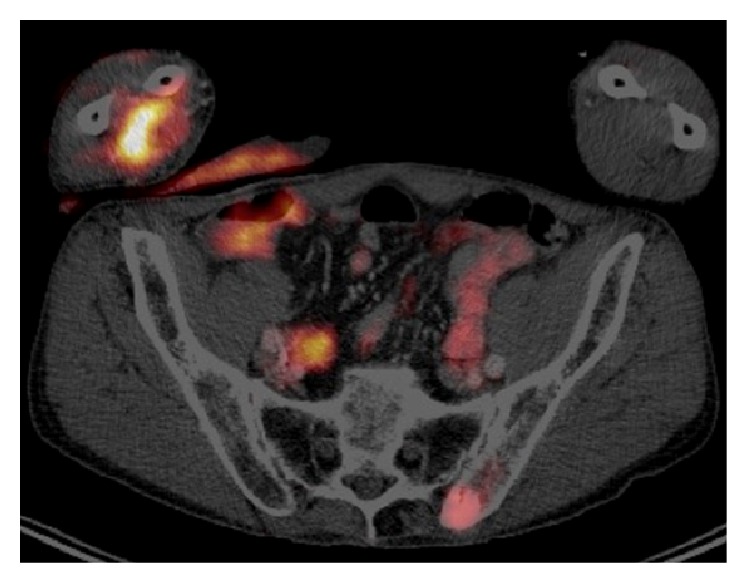
A Pet/CT whole body showed an advanced stage of the cancer, with multiple metastasis in the right forearm and in the left sacroiliac joint and the chest wall.

A Pet/CT whole body (Figure 6) showed a metastatic progression of the cancer, with ectopic lesions to the right forearm, the left sacroiliac joint, and the chest wall.

For a local pain control, a palliative chemotherapy was administered with carboplatin and gemcitabine. Then, 5 cycles of 4 Gy radiotherapy were performed in the forearm (20 Gy).

At 4 months postoperatively, the patient showed a recalcitrant flexion contracture of the long fingers without local pain in the right forearm.

Five months after the surgical excision, the patient died due to the progressive deterioration of the general conditions.

## 3. Discussion

Metastases to the skeletal muscle from distant primary lesions are rare and usually found in case of advanced stage of cancer. Muscles are an unusual site of metastases even though they represent almost 50% of the total body mass [3] and are highly vascularized [4]. Several defensive factors have been proposed to create a hostile environment against metastases such as muscle pH, muscle motion, and ability to remove lactic acid accumulation that plays a role against tumor neovascularity [6–8]. Magee and Rosenthal [9] in 2002 reported that a previously documented local trauma seems to be a risk factor for development of metastasis, probably for an imbalance of local physiological and mechanical factors.

Several studies from autopsies report rates of muscle metastases oscillating from 6% to 17,5% [10, 11]. These data underline the frequency of asymptomatic skeletal muscles metastases, most of all in advanced stage of a malignancy. The majority of these metastases are microscopic lesion undetectable with CT scans and MRI [4, 5].

Skeletal muscles metastases from UCC are, however, very rare and to our best knowledge, only 9 cases have been reported in the English literature [2–6].

The great muscles, such as the erector spinae, psoas, and gluteals, are the most common sites of metastatic involvement (Table 1). Nabi et al. [3] reported metastases to psoas, rectus abdominis, and adductor of the thigh. Doo et al. [5] and Katafigiotis et al. [6] described two metastases to sartorius, while Nagao et al. [4] proposed the case of one in the gluteus maximus.

Ekici et al. [2] described the first case of upper limb metastasis in a deltoid muscle. Our case is the first description of a forearm lesion, with an extensive infiltration of the volar compartments of the forearm, and the first one with a clear functional impairment.

Usually, the clinical presentation of all the reported cases in literature [2–6] is a localized and painful muscular swelling (Table 1). In our case, the patient complained of a bothersome flexion contraction of the fingers with muscular pain, at first assumed as chronic compartment syndrome.

### 3.1. Radiologic Exams

 Ekici et al. [2] emphasize that every localized muscular mass in patients affected previously by transitional cell carcinoma should be considered to be a metastasis and should be investigated.

Skeletal muscle metastases are frequently incidental findings on CT of the chest or abdomen. The CT scans show an increase in ring-shaped enhancement with hypoattenuation of the inner part. MRI findings, although not specific, show low to intermediate signal intensity in the T1-weighted image and uniform high-signal intensity in the T2-weighted image (Table 1).

### 3.2. Treatment

Fine-needle aspiration biopsy with ultrasonographic guidance should be advisable to determine skeletal muscle metastases from UCC.

In our case, we removed the lesion en bloc for diagnosis and treatment, according to Ekici et al. [2] and Katafigiotis et al. [6] that reported good results in terms of pain control with en bloc excision.

Nabi et al. [3] reported that the mean survival rate after receiving chemotherapy was 8 months (min 6–max 12), while Katafigiotis reported a survivorship of 7 months after chemotherapy and radiotherapy.

Chemotherapy with or without the addition of local radiotherapy has been reported as an efficient treatment in skeletal muscle metastases [3–5]. In the study of Nabi et al. [3], all patients had palliative chemotherapy with mitomycin, vincristine, adriamycin, and cyclophosphamide. Ekici et al. [2] used cisplatin, methotrexate, and vinblastine. Katafigiotis et al. [6] administered 6 cycles of gemcitabine and cisplatin. In our case, a carboplatin and gemcitabine protocol has been adopted (Table 1).

Nabi et al. [3] used in two patients a local palliative radiotherapy of 35 Gy. Our patient received 5 cycles of 4 Gy radiotherapy (20 Gy) with good pain control.

Chemotherapy and radiotherapy are usually the preferred palliative treatment method. Surgical resection should be proposed in case of compressive effect of the metastasis, even though en bloc excision can improve pain control in painful muscle metastases [3, 5].

## 4. Conclusion

Muscular localized swelling and lumps in patients with positive clinical history for UCC need to be taken as possible skeletal metastases. Radiological findings and a biopsy may help to study the lesion and to determine the most suitable treatment.

## Figures and Tables

**Table 1:** 

Number	Study	Age	Initial stage of disease	Initial treatment received	Clinical presentation	Radiological presentation: localisation	Treatment received	Follow-up
1	Nabi et al. [3]	65	T3N0M0	Radical cystectomy with sigmoid neobladder	Localized swelling on medial aspect of thigh with pain	CT showing ring-enhancing lesion in the left adductor of the thigh	Palliative chemotherapy	Died at 8 months

2	Nabi et al. [3]	27	T3BN2M0	Radical cystectomy with ileal conduit	Persistent back ache with limp	CT with enlarged swollen left psoas	Palliative chemotherapy with localized radiotherapy	Died at 6 months

3	Nabi et al. [3]	62	T3AN0M0	Radical cystectomy with sigmoid neobladder	Persistent swelling in anterior abdominal wall	CT showing ring-enhancing lesion in left rectus abdominis	Palliative chemotherapy	Died at 12 months

4	Nabi et al. [3]	70	T3BN2M1	None	Left limp with backache	CT with enlarged swollen left psoas	Palliative chemotherapy with localized radiotherapy	Died at 8 months

5	Nabi et al. [3]	36	T3AN1M0	Radical cystectomy with ileal conduit	Pain in the back	CT with swollen left psoas with areas of low attenuations	Palliative chemotherapy	Died at 6 months

6	Doo et al. [5]	45	T2N0M0	Transurethral resection	Left tight pain	MRI: T1 slight high signal, T2 high signal with strong enhancement in left sartorius	Palliative chemotherapy with localized radiotherapy	No data available

7	Katafigiotis et al. [6]	51	T2N0M0	Radical cystectomy and prostatectomy an urinary diversion with ileal conduit	Left tight pain and localized mass	MRI: no data available. Left sartorius	Resection, radiotherapy, chemotherapy	Alive at 7 months

8	Ekici et al. [2]	41	T3N0M0	Chemotherapy	Fixed mass on the deltoid muscle, painless	MRI: no data available. Right deltoid muscle	Resection, chemotherapy	Died at 9 weeks

9	Nagao et al. [4]	63	T3N1M1	Chemotherapy and radiotherapy	Swelling localized on the right gluteus	Ct with slight enhancement, gluteus maximus	No data available	No data available

10	Fusetti, Guidi, Lucchina	76	T3N0M0	Radical cystectomy with ileal conduit	Painful flexion contracture of the proximal interphalangeal joints of the fingers	MRI: T2 high signal with strong enhancement of the flexor digitorum superficialis and profundus	Resection, chemotherapy	Died at 5 months
